# Paediatric chronic suppurative otitis media in rural Rwanda: Prevalence and parental practices

**DOI:** 10.4102/jcmsa.v2i1.77

**Published:** 2024-08-20

**Authors:** Gratien Tuyishimire

**Affiliations:** 1 Department of Otolaryngology, University Teaching Hospital of Kigali, Kigali, Rwanda

**Keywords:** chronic otitis media, otorrohea, tympanic membrane pathology, traditional medicine, health practice and beliefs, health literacy

## Abstract

**Background:**

Chronic suppurative otitis media (CSOM) is a common paediatric ear disease in developing countries, typically resulting from an acute otitis media (AOM) that is not promptly diagnosed or treated. Home remedies using herbal medicine in rural villages may contribute to treatment delays and complications.

**Methods:**

A cross-sectional study of children from three rural schools was conducted. Children between 6 and 14 years of age were randomly selected and screened by the lead author for CSOM between January and February 2015 by history and physical examination, including otoscopy. Parents of children diagnosed with CSOM were asked additional questions regarding their understanding of this clinical entity.

**Results:**

A total of 461 children with a mean age of 9.8 years were screened and 17 were diagnosed with CSOM (3.6%). The majority (*n* = 10/17, 59%) of parents reported no understanding of the aetiology of their children’s ear symptoms and the remainder (*n* = 7, 41%) reported other incorrect aetiologies. Traditional home remedies such as instilling fruit juice are still common in this community. Most parents confirmed to have previously used traditional remedies at least once to treat their children for CSOM.

**Conclusion:**

Approximately one in 25 school-aged children in rural Rwanda had CSOM at the time of this study. Parental knowledge about the causes, treatments and complications of CSOM is limited.

**Contribution:**

This study provided insights into the prevalence of CSOM in school-aged children in the Rwandan district of Bugesera and explored current beliefs and practices among parents of children with CSOM.

## Introduction

Chronic suppurative otitis media (CSOM) is characterised by a chronic infection of the middle ear with a perforated tympanic membrane resulting in at least 2 weeks of otorrhoea.^1,3^ It is a common paediatric ear disease, classically resulting from acute otitis media (AOM) that is not promptly diagnosed or adequately treated. Chronic suppurative otitis media in childhood is associated with complications ranging from moderate conductive hearing loss (which may affect the development of speech, language, cognition and school performance) to life-threatening suppurative complications and death.^2^

Worldwide, over 90% of the burden of disease of CSOM is borne by developing countries in Africa, South-east Asia, Western Pacific areas, and the Pacific rim.^3^ Although the prevalence of CSOM has declined worldwide in recent decades because of improvements in housing conditions, personal hygiene and antimicrobial therapy, poor health literacy remains a major risk factor for poor outcomes in certain impoverished rural communities.^3,4,5^ This study measured the prevalence of CSOM in school-aged children in the rural district of Bugesera in Rwanda and explored the local parental understanding of this disease.

## Research methods and design

A cross-sectional study was conducted in public primary schools in the Bugesera district of Rwanda from January 2015 to February 2015. The target study groups were children between 6 years and 14 years of age and parents of students with CSOM. A Fisher’s sample size calculation was used to determine the number of students who needed to be screened to determine the prevalence of CSOM in this community at the 95% confidence level and 5% precision (assuming maximum variability *p* = 0.5, minimum sample size *n* = 423), where approximately 110% of this number was used as the target enrolment in this study. Three rural schools were randomly selected from 81 primary schools in the district: Mayange (A) Primary School, Muyenzi Primary School, and Nyirarukobwa Primary School. The number of students sampled at each school was proportional to their student populations. At each site, the number of participants sampled from each of the six class levels was proportional to the number of students in each class. Students were enrolled in alphabetical order using class registers until the number allocated to each class was reached. Children whose parents did not gave written informed consent for the study and children who refused ear examinations were excluded.

Clinical histories and physical examinations including otoscopy were performed by local otolaryngologists and senior otolaryngology residents helped in data recording. During physical examinations, cerumen was evacuated where possible using ear curettes. If not possible, phenol glycerine ear drops were given for 5–7 days to soften impacted wax before cerumenectomy. The criteria for diagnosing CSOM included history of ear discharge for at least 2 weeks and tympanic membrane perforation and/or cholesteatoma on otoscopic examination. Children diagnosed with CSOM were treated and/or referred for further management.

After students were screened for CSOM, the parental guardians of children diagnosed with CSOM were contacted at home for one-on-one interviews regarding their beliefs and practices surrounding this clinical entity. Interview questions explored their medical knowledge, beliefs and existing practices regarding their children’s otologic disease.

### Ethical considerations

The Institutional Review Board at a Rwandan University approved the research and granted ethical clearance for this study. Approval was also obtained from the district governmental authorities. Written consent was obtained for all patients and legal guardians and all patient information was handled confidentially and de-identified during analysis.

## Results

A total of 461 children were screened for CSOM. Participants had a mean age of 9.8 years ± standard deviation 2.4. Half of the participants (51.6%) were female. Seventeen cases of CSOM were diagnosed, returning an overall prevalence rate of 3.7% (Table 1). Two patients had atticoantral CSOM with cholesteatoma and the remaining 15 patients were found to have tubotympanic CSOM. Three patients had active ear discharge, but no other complications were found.

**TABLE 1:** Prevalence of chronic suppurative otitis media in school-aged children in rural Rwanda.

Sex	Children screened		Diagnosed with chronic suppurative otitis media	
	*n*	%	*n*	%
Female	238	52	8	3
Male	223	48	9	4

**Total**	**461**	**100**	**17**	**4**

Parents of children with CSOM (*n* = 17) were interviewed. The mean age of parental guardians was 39 years. When asked about the aetiology of CSOM, 10 parents out of 17 (59%) mentioned a chronic draining ear disease (known in the local dialect as ‘*Umuhaha*’), which commonly starts in early childhood (younger than 5 years of age). Five parents (29%) reported that this illness is more likely to occur in school-aged children. Parents demonstrated poor understanding of the causes of CSOM, citing aetiologies such as transmission and/or contamination from other infected children or poisoning (Table 2).

**TABLE 2:** Parental understanding of the aetiology of paediatric chronic suppurative otitis media.

Aetiology	*n*	%
Unknown	10	59
Water in the ear	2	12
Poisoning	2	12
Contamination from other children	1	6
Foreign body	1	6
Tears flowing into the ear when crying	1	6

**Total**	**17**	**100**

Among the respondents, 11 parents (65%) had never heard of dry ear precautions. Only five parents (29%) acknowledged the relationship between persistent or recurrent ear drainage and water entry into the ear. If their child had an actively draining ear, 10 parents out of 17 (59%) reported they would take the child to a healthcare centre, while the remainder reported they would try alternative therapies such as over-the-counter medications, instilling plant juice into the ear, or irrigating the ear (Table 3).

**TABLE 3:** First-line treatment to treat paediatric chronic suppurative otitis media according to interviewed parents.

Treatment	*n*	%
Seeking care at a health centre	10	59
Buying medicine at the pharmacy	2	12
Instilling plant juice	3	18
Seeing a traditional healer	1	6
Irrigating with water	1	6

**Total**	**17**	**100**

Thirteen of 17 parents (76.5%) reported having delayed seeking medical attention for a child with a draining ear. Trust in traditional medicine was the main reason for this delay reported by five parents. Sixteen of 17 parents reported having applied water or traditional medicines into their child’s ear at least once to treat their CSOM (Table 4). Fifteen out of 17 parents (88%) were not aware of the role of surgery in the treatment of CSOM and only one parent believed it could be necessary.

**TABLE 4:** Traditional medicines used by parents to treat paediatric chronic suppurative otitis media.

Traditional medicine	*n*	%
Plant juice	10	59
Cold oil drops	4	24
Cow’s milk	1	6
Chameleon tail	1	6
None	1	6

**Total**	**17**	**100**

Parental knowledge about the complications of CSOM was also limited. Only nine out of 17 (52%) listed deafness as a major complication. Only three parents (16%) anticipated persistent drainage in cases where no treatment is provided. Two parents out of 17 (11.8%) believed the disease can spread to other organs, especially the contralateral ear. None of the parents believed chronic ear infection could cause death when there is an intracranial spread.

## Discussion

This study found the prevalence of CSOM in school-aged children in rural Rwanda to be 3.7%, which is lower than the prevalence among school-aged children in rural Tanzania (9.4%),^6^ Yemen (7.4%), South India (6%)^7^ or Malawi (5.4%).^8^ However, this is higher than the prevalence of CSOM in prior studies of urban primary schools in the Rwandan capital of Kigali (2.1%),^9^ reflecting a disparity found in other studies whereby children in rural areas may have more poverty and hygiene-associated risk factors for CSOM^10^ compared with those in urban living environments.^6^

Parental knowledge of the causes and treatments of CSOM was very poor in this study, similar to previous reports of lay-understanding of this clinical entity among families of children with this disease in developing countries.^1,7^ This poor level of health literacy may result from limited access to primary care providers and limited access to health education delivered through technology and media channels in these communities. Even though a high proportion of parents in this study stated they would take the child with ear complaints to a health centre, nearly all questioned parents reported having tried a traditional medicine at least once to treat their child’s otorrohea. There are similarities in this study’s reports of the traditional medications used to treat draining ears with studies of other communities across sub-Saharan Africa. Our community shares the same practices as Kenyans^11^ and Nigerians^12^ who also use plant juice drops or animal products such as cow’s milk drops in children’s ears. Kenyans also reported using chicken soup or fat, and Nigerians reported using goats nasal discharge and honey. The use of commercial products in draining ears such as cold cooking oil in our study was similar to reports of the use of gun oil in Kenya. However, this study did not find any cases of religious practices being used to treat ear disease, unlike the usage of holy water drops in Nigeria^12^ or prayer as reported in Kenya.^11^

The common reliance on traditional remedies may result from a lack of knowledge about the efficacy of modern medical treatments and a limited understanding of the potential negative impacts of these home therapies that introduce foreign substances into the ear. However, it is possible that some traditional medicines might have anti-inflammatory properties that may be useful in the treatment of otologic diseases. In this study, parents were unaware that surgery was a possible treatment for CSOM. This likely reflects poor access to otolaryngologic services in Rwanda, similar to many other countries across Africa.^13^

Despite the possible life-threatening complications associated with CSOM,^2,14,15^ knowledge about these complications was very poor among parents interviewed in this study. While more than half accurately cited deafness as a possible complication, only two parents understood the possibility of the disease to spread to neighbouring structures, such as the brain. At the same time, no parent believed that CSOM-related complications could lead to death. This could be one reason families are not motivated to seek medical attention in these rural communities. Future health education interventions are needed to increase awareness about the short and long-term consequences of untreated paediatric CSOM.

### Limitations

This study sampled one rural district among 27 districts in Rwanda, with findings that may or may not be generalisable to other districts or countries. As there were only a small number of cases of CSOM, a limited number of parents were interviewed, and their responses may not be reflective of all others in the community. Lastly, this is a cross-sectional study of CSOM at a single timepoint and the prevalence of the disease may vary across time or seasons.

## Conclusion

In this study, one in every 25 children in the rural Rwandan district of Bugesera was diagnosed with CSOM. Parental knowledge about CSOM is very limited and traditional medical practices to treat chronically draining ears with oils or juices are still common. Efforts to raise awareness about the prevention, diagnosis, and treatment of CSOM are needed.
